# Intermolecular interactions between β-cyclodextrin and water

**DOI:** 10.1039/d1ra03960a

**Published:** 2021-07-16

**Authors:** Tianxiang Guo, Lingfeng Kong, Junpeng Xu, Yuhan Geng, Runan Zhang, Yuanfeng Pan, Huining Xiao

**Affiliations:** Hebei Key Lab of Power Plant Flue Gas Multi-Pollutants Control, Department of Environmental Science and Engineering, North China Power University Baoding 071003 PR China; MOE Key Laboratory of Resources and Environmental Systems Optimization, College of Environmental Science and Engineering, North China Electric Power University Beijing 102206 PR China; School of Chemistry and Chemical Engineering, Guangxi University Nanning 530004 PR China; Department of Chemical Engineering, University of New Brunswick Fredericton E3B 5A3 Canada hxiao@unb.ca

## Abstract

This study focused on demonstrating the intermolecular interactions between β-cyclodextrin and water, with the aim to better understand the transfer of small molecules to β-cyclodextrin. The intermolecular interaction strength between β-cyclodextrin and water was analyzed using different methods such as the dynamic adsorption of water, the TG-DSC of β-cyclodextrin and molecular modeling employing MM2 force field calculations. The experiments for the adsorption of water on β-cyclodextrin was aimed to systematically investigate the adsorption characteristics, such as adsorption capacity, adsorption rate, adsorption heat and activation energy, influenced by the adsorption temperature and vapor pressure of water. The results indicated that the water adsorption on β-cyclodextrin is an exothermic process. The hysteresis loop type in the adsorption isotherms at multiple temperatures indicated that water adsorption is not purely a traditional physical adsorption due to the existence of structure effects such as the cavity effect and hydrogen bonding. The activation energy during water adsorption was 7.4 kJ mol^−1^. However, the activation energy during water desorption was in the range of 35–45 kJ mol^−1^, which decreased with an increase in the amount of water adsorbed. This indicated that water adsorption is much easier than water desorption from β-cyclodextrin and that water desorption is more difficult with a small amount of adsorbed water compared with a large amount of adsorbed water. Subsequently, the obtained average intermolecular interaction strength between β-cyclodextrin and water under the experimental conditions was 67.5 kJ mol^−1^ (water), which was verified by DSC.

## Abstract 2

This study focused on demonstrating the intermolecular interactions between β-cyclodextrin and water, with the aim to better understand the transfer of small molecules to β-cyclodextrin.

## Graphical abstract

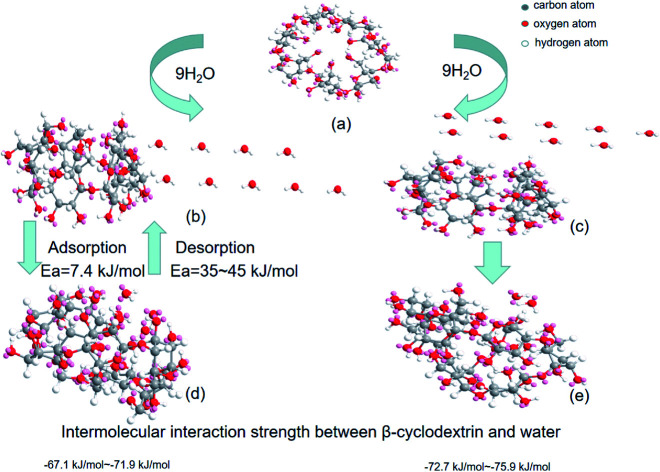

## 1. Introduction

Cyclodextrins (CDs) are cyclic oligomers of d-(+)-glucopyranose units linked through α-1,4-glycoside bonding, which include α-cyclodextrin (six d-galactose groups), β-cyclodextrin (seven d-galactose groups) and γ-cyclodextrin (eight d-galactose groups).^1^ Considering the nontoxic effect and molecular recognition ability of CDs as functional compounds practically,^2–5^ they have been widely applied in many industries such as the food industry, environmental protection, biotechnology and cell biology.^6^ Among CDs, β-cyclodextrin is the most widely used cyclodextrin because of its hydrophilic exterior (primary and secondary hydroxyls) and hydrophobic molecular cavity, which can provide hydrophobic binding sites for inorganic ions and guest organic molecules^7,8^ by forming a relatively stable structure^9,10^ based on intermolecular interactions such as van der Waals force. Moreover, the hydrophilic exterior of its cavity makes β-cyclodextrin exhibit good interactions with hydrophilic substances, and thus it has been used in the fields of drug delivery, pollutant removal and synthesis of new materials.^11–15^ The role of water is important to accurately understand the transfer of small molecules to β-cyclodextrin and the intermolecular interactions between β-cyclodextrin and other species besides water. Therefore, it is necessary to accurately investigate the intermolecular interactions between β-cyclodextrin and water.^16,17^

To demonstrate the intermolecular interactions between β-cyclodextrin and water, the adsorption behaviors of water on β-cyclodextrin were systematically investigated, and the adsorption characteristics such as adsorption capacity, adsorption rate, equivalent adsorption heat and activation energy influenced by the adsorption temperature and vapor pressure of water were elucidated. The equivalent adsorption heat based on the Clausius–Clapeyron equation represents the average strength of the thermal effect under experimental conditions.^18–22^ However, water adsorption on β-cyclodextrin is a complex process.^23,24^ Although there are a few studies about the existing conditions of water inside the cyclodextrin molecule,^25,26^ such as seven molecules of water in the cyclodextrin cavity, the various thermodynamic and dynamic parameters such as the activation energy and interaction strength during water adsorption on β-cyclodextrin have been rarely reported. Herein, different methods including water dynamic adsorption, TG-DSC of β-cyclodextrin and molecular modeling employing MM2 force field calculations were used to investigate thermodynamic and dynamic parameters such as the activation energy of water adsorption on β-cyclodextrin and the interaction strength between β-cyclodextrin and water.

## 2. Methods

β-Cyclodextrin (BR) was purchased from Beijing Shuangxuan Microbe Culture Medium Products Factory and used for dynamic water adsorption. The water adsorption tests were carried out using the BSD-VS system (Beishide Instrument Technology Co., Ltd., Beijing). Considering water condensation under saturated humidity at a given temperature, 95% relative humidity was selected as the end pressure of adsorption. Therefore, the dynamic water adsorption was performed at a relative humidity of 10–95% at 283 K, 298 K and 313 K. Prior to the adsorption experiments, all the as-prepared β-cyclodextrin samples (50–100 mg) were dried at 473 K for 8 h.

To investigate the desorption of water from β-cyclodextrin, the dehydration process of β-cyclodextrin stored in the ambient environment was analyzed *via* TG-DSC using a Mettler LF/1100 (TA Instruments, Sweden) under nitrogen with a flow rate of 100 mL min^−1^ in the temperature range of 303 K to 473 K at a heating rate of 5 K min^−1^, 10 K min^−1^, 15 K min^−1^ and 20 K min^−1^. Below 473 K, β-cyclodextrin would not chemically decompose, and thus the dehydration process of β-cyclodextrin reflects the desorption of water from β-cyclodextrin. The dehydration of β-cyclodextrin at the heating rate of 5 K min^−1^ was performed four times. Then the dehydration rate was expressed as the first derivative of mass loss with temperature due to the linear relationship between temperature and time.

Molecular modeling employing MM2 force field calculations based on the ChemOffice suite of programs was performed to investigate the intermolecular interactions between β-cyclodextrin and water. The initial configuration of β-cyclodextrin came from the template database (supramolecules) of the ChemOffice suite of programs. Also, the optimum structure of the initial configuration based on the minimum energy optimization (minimum RMS gradient: 0.01) was used to theoretically investigate the intermolecular interactions between β-cyclodextrin and water. The molecular modeling employing MM2 force field calculations was related to the minimum energy and molecular dynamics.

## 3. Results and discussion

### 3.1 Adsorption isotherms

**Fig. 1:**
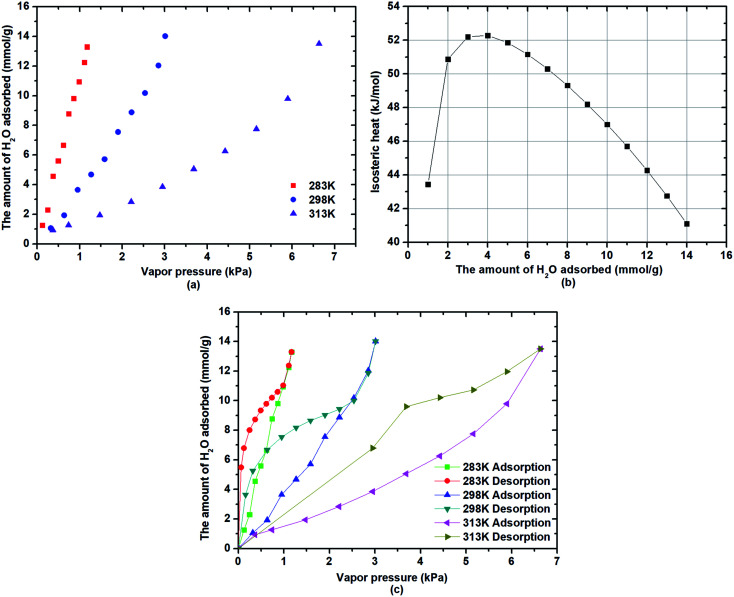
Characteristics of water adsorption: capacity changes (a), isosteric heat (b) and adsorption isotherms (c).

The water adsorption on β-cyclodextrin in the pressure range of 0–8 kPa was measured at 283 K, 298 K and 313 K, and the results are presented in Fig. 1. As seen in Fig. 1(a), the water adsorption capacity increased with an increase in vapor pressure, whereas it increased with a decrease in the adsorption temperature. The water adsorption on β-cyclodextrin conformed to the type III adsorption isotherm. For instance, when the adsorption temperature was 283 K, the adsorption capacity for water was 5.7 mmol g^−1^ at the vapor pressure of 0.5 kPa, and then increased to 11.1 mmol g^−1^ at a vapor pressure of 1 kPa. However, when the vapor pressure was 1 kPa, it decreased to 3.8 mmol g^−1^ at 298 K and 1.5 mmol g^−1^ at 313 K. The increasing tendency of the adsorption capacity with an increase in vapor pressure indicates that the pressure exhibited a positive effect on the water adsorption on β-cyclodextrin. Also, an increase in pressure is beneficial for water uptake. Then, the decreasing tendency of the adsorption capacity with an increase in the adsorption temperature indicates that the adsorption of water on β-cyclodextrin is an exothermic process. The temperature exhibited a negative effect on water adsorption. Also, a decrease in temperature is conducive to water uptake.

1 (Δln *P*/Δ*T*)_*q*_ = *Q*/*RT*^2^

where *P* represents the adsorption equilibrium pressure, *T* represents the adsorption temperature, *Q* represents the adsorption heat, *R* is the gas constant, and *q* is the amount of water adsorbed.

Combining the Clausius–Clapeyron relation (1) with the ideal gas state equation, the isosteric heat of water adsorption on β-cyclodextrin was obtained, as shown in Fig. 1(b), which varied in the range of 40–53 kJ mol^−1^. Moreover, it depended on the amount of water adsorbed, showing a tendency of increasing firstly, and then decreasing with an increase in the amount of water adsorbed. The isosteric heat of more than 40 kJ mol^−1^ indicated that the water adsorption on β-cyclodextrin was different from the traditional physical adsorption with an isosteric heat below 20 kJ mol^−1^, which may be attributed to the existence of a structure effect such as hydrogen bonding due to the large amount of hydrophilic hydroxyl groups in β-cyclodextrin. In addition, as shown in Fig. 1(c), the adsorption curve is obviously inconsistent with the desorption curve at a given adsorption temperature, accompanied by an obvious hysteresis loop, regardless of the adsorption temperature. This may have resulted from the special molecular cavity structure of β-cyclodextrin. This phenomenon is reflected in the fact that water desorption from β-cyclodextrin was more difficult than water adsorption.

### 3.2 Adsorption rate

**Fig. 2:**
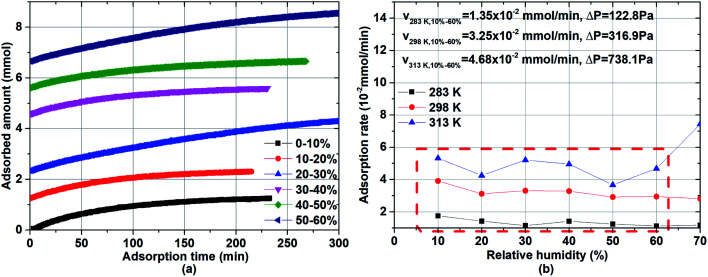
Relationship between the adsorption amount and adsorption time: (a) adsorption capacity at 283 K and (b) adsorption rate.

To investigate the rate of water adsorption on β-cyclodextrin, the changes in water adsorption *vs.* adsorption time at different adsorption temperatures and pressure (relative humidity) ranges were analyzed, and the results are shown in Fig. 2. As shown in Fig. 2(a), the amount of water adsorbed at given adsorption temperature such as 283 K exhibited a tendency of increasing rapidly at first, and then slowly increasing with an increase in adsorption time at a given pressure (relative humidity). The nonlinear relationship between the adsorption amount and adsorption time indicates that the adsorption rate, as determined from the slope of the adsorption amount *vs.* adsorption time, decreased with an increase in adsorption time at a given adsorption temperature and pressure (relative humidity). This is because the adsorption rate at a given adsorption temperature and pressure (relative humidity), which can be described by eqn (2) with condition (3), is affected by both the dynamic item *k*_*i*_*P*_*i*_ and thermodynamic term (1 − *P*_*i*_(*q*)/*P*_*i*_).2
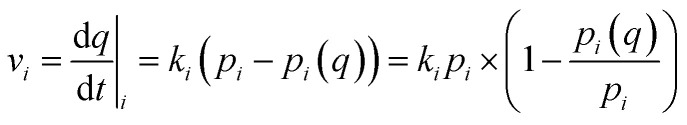
3
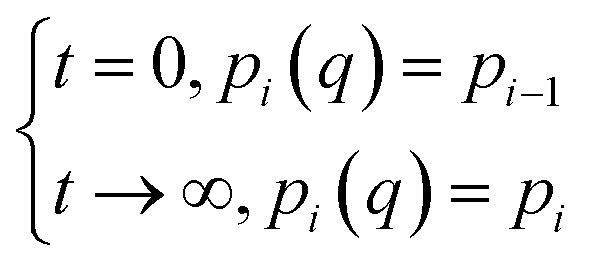
where *P*_*i*_(*q*) represents the adsorption equilibrium pressure with the adsorbed amount *q*, *P*_*i*_ represents the constant pressure of the adsorption stage *i*, *P*_*i*−1_ represents the constant pressure of adsorption stage *i* − 1, and *k*_*i*_ represents the rate constant of the adsorption stage *i* at a given adsorption temperature.

When the adsorbed amount *q* increased, the equilibrium pressure *P*_*i*_(*q*) increased from pressure *P*_*i*−1_. In the initial adsorption stage *i*, the equilibrium pressure *P*_*i*_(*q*) was close to pressure *P*_*i*−1_, and thus the adsorption rate mainly depended on the dynamic item *k*_*i*_*P*_*i*_ and was higher compared with the subsequent adsorption. In the final adsorption stage *i*, the equilibrium pressure *P*_*i*−1_ was gradually close to pressure *P*_*i*_, and thus the adsorption rate mainly depended on the thermodynamic term (1 − *P*_*i*_(*q*)/*P*_*i*_) and decreased with an increase in adsorption time until it was close to zero. To investigate the activation energy during water adsorption, the adsorption rate for a given pressure *P*_*i*_ at a given adsorption temperature was obtained according to the initial rate method, together with the driving force of *P*_*i*_ − *P*_*i*−1_ (eqn (4)). It was calculated using the slope of the adsorption amount *vs.* adsorption time based on the top 10 adsorption data. Also, the obtained adsorption rates are shown in Fig. 2(b).4
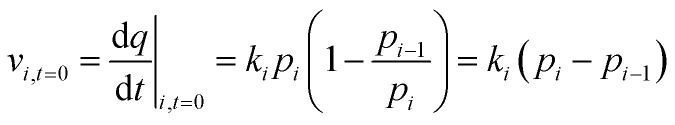
5
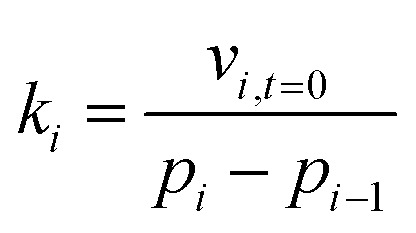
6
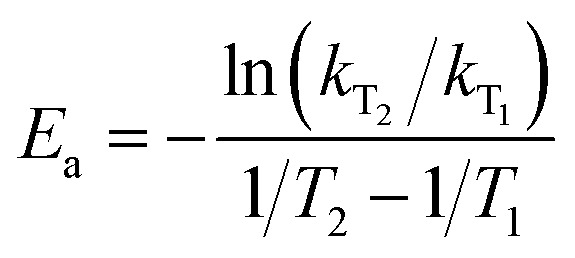


As shown in Fig. 2(b), the obtained adsorption rate increased with an increase in adsorption temperature, regardless of the relative humidity range. Considering the unstable adsorption at high vapor pressure (relative humidity), the average adsorption rate under the relative humidity range of 10–60% was selected to calculate the adsorption rate, which was 1.35 × 10^−2^ mmol min^−1^ at 283 K, 3.25 × 10^−2^ mmol min^−1^ at 298 K and 4.68 × 10^−2^ mmol min^−1^ at 313 K, with the pressure driving force of 122.8 Pa, 316.9 Pa and 738.1 Pa, respectively. Then the adsorption rate constant *k*_*i*_ at a given adsorption temperature was obtained from eqn (5). According to eqn (6), the activation energy was calculated based on the adsorption rate constants at different adsorption temperatures *T*_1_ and *T*_2_, and the results are listed in Table 1.

**Table 1:** Calculation of activation energy

Temperature, K	283	298	313
Adsorption rate constant *k*, 10^−5^ mmol min^−1^ Pa	10.64	9.12	7.88
ln(k)	−9.15	−9.30	−9.45
Apparent activation energy, kJ mol^−1^	7.4		
Pre-exponential factor, mmol min^−1^ Pa	4.7 × 10^−6^		
Square. *R*^2^	0.9999		

As seen from Table 1, the apparent rate constant of water uptake decreased with an increase in the adsorption temperature, which changed from 10.64 × 10^−5^ mmol min^−1^ Pa at 283 K to 9.12 × 10^−5^ mmol min^−1^ Pa at 298 K and 7.88 × 10^−5^ mmol min^−1^ Pa at 313 K. Subsequently, according to eqn (6), the activation energy of water adsorption on β-cyclodextrin was obtained from the slope of ln *k vs.* 1/*T*, which was 7.4 kJ mol^−1^. The low activation energy indicates that water adsorption on β-cyclodextrin is easy.

### 3.3 Water desorption from β-cyclodextrin

**Fig. 3:**
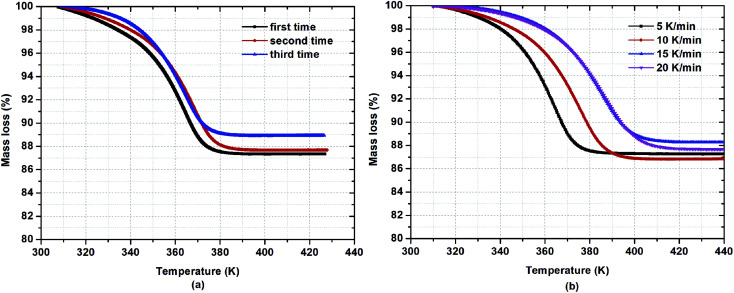
Mass loss of β-cyclodextrin at a heating rate of 5 K min^−1^ (a) and different heating rates (b).

To investigate the water desorption characteristics from β-cyclodextrin in the ambient environment, the thermogravimetric analysis of β-cyclodextrin was performed based on the TG tests in a heating rate range of 5–20 K min^−1^. The related results are presented in Fig. 3. According to the mass loss shown in Fig. 3(a), the water content of β-cyclodextrin between 308 K and 313 K varied in the range of 11.1–12.6% based on three parallel tests at a heating rate of 5 K min^−1^. The average water content was 12.0% with an error of 1%. According to the average water content of β-cyclodextrin, the mole *n* of water adsorbed by per mole of β-cyclodextrin could be obtained based on formula (7).7
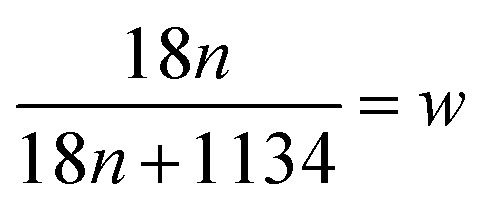
where *w* represented the water content of β-cyclodextrin.

According to formula (7), the obtained mole *n* of water adsorbed by per mole of β-cyclodextrin was 8.6 based on three parallel tests at a heating rate of 5 K min^−1^. The average end temperature for mass loss was considered as 385 K, with a mass change of less than 0.1% compared with the final mass of β-cyclodextrin. Considering the complete desorption of water, the heating time for the dehydration of β-cyclodextrin was determined to be at least 16 min when the heating rate was set at 5 K min^−1^.

To further reveal the characteristics of water desorption, the mass loss of β-cyclodextrin at different heating rates was analyzed based on Fig. 3(b). As seen in Fig. 3(b), the water content of β-cyclodextrin varied in the range of 11.8–13.2% based on four tests at heating rates of 5 K min^−1^, 10 K min^−1^, 15 K min^−1^ and 20K min^−1^, respectively. The average value of water content was 12.5% with an error of 1%. According to formula (6), the mole of water adsorbed by per mole of β-cyclodextrin was considered to be 9. Therefore, the number of moles of water adsorbed by per mole of β-cyclodextrin in the ambient environment was between 8.5 and 9.

Meanwhile, as also shown in Fig. 3(b), when the heating rates were 5 K min^−1^, 10 K min^−1^, 15 K min^−1^ and 20 K min^−1^, the end temperatures were determined to be 385 K, 406 K, 420 K and 425 K, respectively. The end temperature increased with an increase in heating rate, accompanied by a decrease in the heating time for the complete desorption of water, which was 16.8 min, 9.6 min, 7.4 min and 6.8 min, respectively. This indicates that the dehydration process of β-cyclodextrin needed more time at a relatively low heating rate for the complete removal of water from β-cyclodextrin.

### 3.4 Desorption rate

**Fig. 4:**
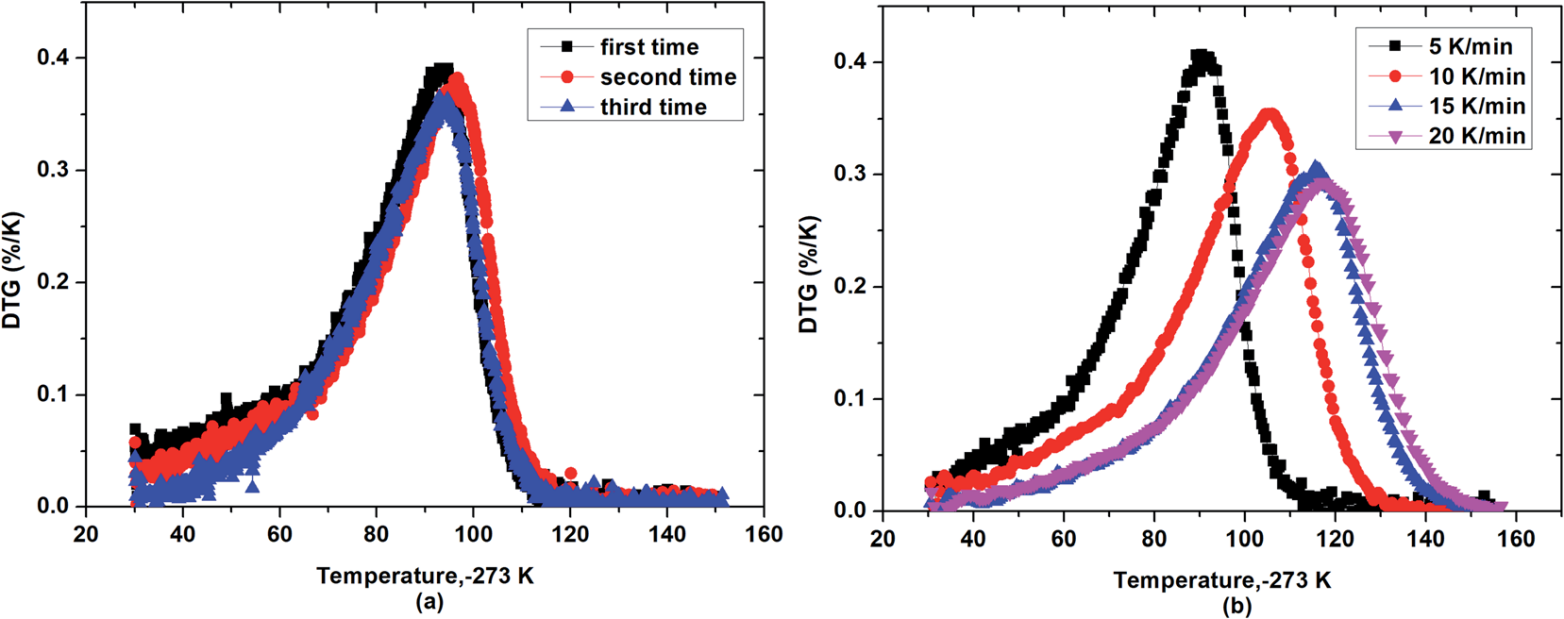
Mass loss rate curves of β-cyclodextrin at the heating rate of 5 K min^−1^ (a) and different heating rates (b).

As also seen in Fig. 3, based on the slope of mass loss *via* temperature, β-cyclodextrin seemed to exhibit a lower desorption rate of water at the initial and final time than that at the middle time during the dehydration process, indicating that the desorption rate of water from β-cyclodextrin was different at different temperatures and there was a maximum mass loss rate existing during the dehydration process of β-cyclodextrin. Thus, to verify this phenomenon, Fig. 4 presents the mass loss rates of β-cyclodextrin at the same heating rate and different heating rates. As shown in Fig. 4(a), for a given heating rate, the mass loss rate of β-cyclodextrin exhibited an increasing tendency initially, and then decreased with an increase in temperature. Also, all the samples had one almost identical peak of mass loss rate at the heating rate of 5 K min^−1^, and the observed peak temperatures with maximum mass loss rate of three parallel samples were almost coincident, which was about 365 K, with an error of ±0.7 K. As seen in Fig. 4(b), the observed peak value of mass loss rate decreased when the heating rate increased, which was 0.41%/K at a heating rate of 5 K min^−1^, 0.35%/K at a heating rate of 10 K min^−1^, 0.31%/K at a heating rate of 15 K min^−1^ and 0.29%/K at a heating rate of 20 K min^−1^, with the peak temperatures at 365 K (92 °C), 379 K (106 °C), 389 K (116 °C) and 390 K (117 °C), respectively.

### 3.5 Activation energy of water desorption

To determine the activation energy of water desorption from β-cyclodextrin, the related mass loss was redefined as eqn (8) and the related mass loss rate was defined as eqn (9). Then the activation energy was obtained according to eqn (10) from the curve slope of ln(d*α*/d*t*) *vs.* 1/*T* based on the isoconversion method. The results are shown in Table 2. As shown in Table 2, the mass loss rate d*α*/d*t* at a given heating rate exhibited a trend of increasing initially, and then decreased with an increase in the conversion rate *α*. At given conversion rate *α*, the mass loss rate d*α*/d*t* increased with an increase in the heating rate, accompanied by an increase in temperature. Then the maximum mass loss rate appeared when the conversion rate *α* was near 0.7. According to eqn (10), the obtained activation energy of water desorption from β-cyclodextrin varied in the range of 37–55 kJ mol^−1^. This is much higher than that of the adsorption stage (7.4 kJ mol^−1^), which indicates that water adsorption on β-cyclodextrin is much easier than water desorption.8
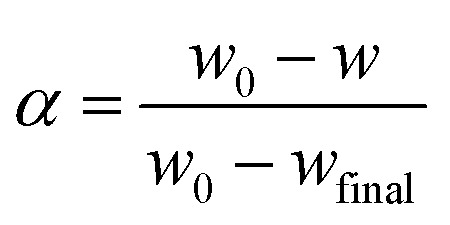
9
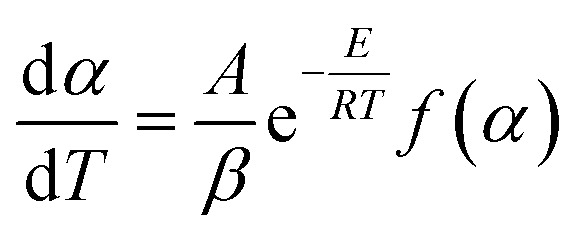
10
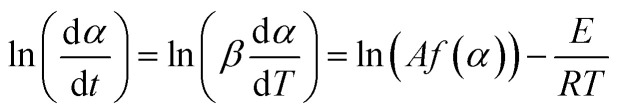


**Table 2:** Calculated activation energy

*α*		Heating rate, K min^−1^				Fitting parameters		
		5	10	15	20	Activation energy *E*, kJ mol^−1^	ln(*Af*(*α*))	*R* ^2^
0.1	*T*, K	329.3	336.5	350.0	348.3	48.3	5.6	0.9196
	d*α*/d*t*, s^−1^	0.5	0.9	1.4	1.7			
0.2	*T*, K	340.9	350.5	363.0	363.0	54.5	7.6	0.9391
	d*α*/d*t*, s^−1^	1.0	1.5	2.6	3.6			
0.3	*T*, K	347.6	359.5	370.5	371.7	49.5	5.9	0.9760
	d*α*/d*t*, s^−1^	1.4	2.4	3.7	4.6			
0.4	*T*, K	352.6	365.5	376.5	377.7	47.8	5.4	0.9825
	d*α*/d*t*, s^−1^	1.8	3.1	4.8	5.8			
0.5	*T*, K	356.8	370.0	381.5	383.0	46.4	4.9	0.9835
	d*α*/d*t*, s^−1^	2.2	3.8	5.7	7.0			
0.6	*T*, K	360.5	374.5	385.5	387.7	44.9	4.4	0.9881
	d*α*/d*t*, s^−1^	2.6	4.3	6.3	7.7			
0.7	*T*, K	363.4	378.0	389.5	391.7	42.9	3.7	0.9900
	d*α*/d*t*, s^−1^	2.7	4.5	6.5	7.8			
0.8	*T*, K	366.8	382.0	393.5	396.3	39.0	2.2	0.9922
	d*α*/d*t*, s^−1^	2.6	4.2	5.8	7.0			
0.9	*T*, K	370.9	386.5	398.5	402.3	37.4	1.1	0.9869
	d*α*/d*t*, s^−1^	1.7	3.0	3.8	4.4			

### 3.6 Enthalpy change of water desorption

**Fig. 5:**
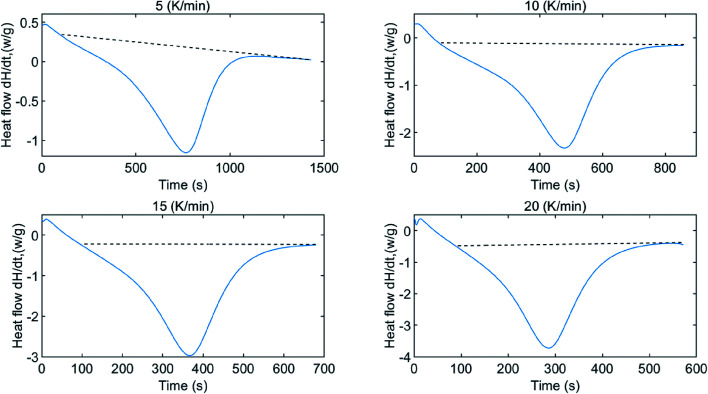
Heat flow of water desorption from β-cyclodextrin at different heating rates.

To determine the enthalpy change of water desorption from β-cyclodextrin, DSC tests at different heating rates were performed, and the results are shown in Fig. 5. As shown in Fig. 5, based on the negative value, the heat flow exhibited a trend of decreasing initially, and then increased with time, indicating that the dehydration process of the sample is an endothermic process. The absolute value of heat flow seemed to vary with the change of mass loss rate. The enthalpy change of dehydration could be calculated using eqn (11). Considering the base line modification, according to eqn (11), the enthalpy change of water desorption from β-cyclodextrin was determined to be in the range of 62.9–71.6 kJ mol^−1^ (water), with an average value of 67.5 kJ mol^−1^ (water) and maximum error of 6 kJ mol^−1^ (water).11
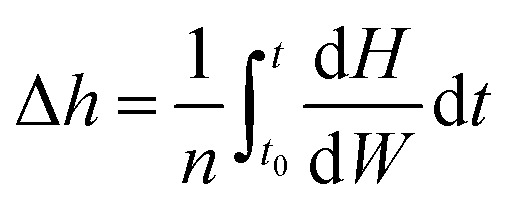


### 3.7 Model validation

**Fig. 6:**
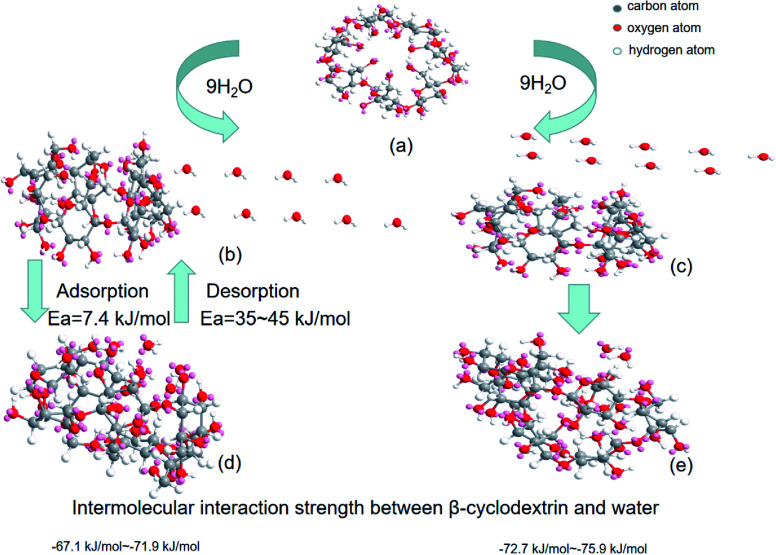
Spatial configuration during molecular dynamics: (a) β-cyclodextrin, (b) initial configuration of β-cyclodextrin with nine water molecules on the cavity side, (c) initial configuration of β-cyclodextrin with nine water molecules inside the cavity, and (d) and (e) spatial configurations after molecular dynamics simulation.

To verify whether the above energy calculation was consistent with the expectation, MM2 force field calculations based on the ChemOffice suite of programs were performed. Firstly, the spatial configuration of β-cyclodextrin, as shown in Fig. 6(a) was set and the static energy was obtained by MM2 force field calculation.^28,29^ During the simulation, molecular dynamics and minimal energy model fitting were used to check whether a certain water molecule could enter the cyclodextrin cavity based on the modification of the relative positions between cyclodextrin and the water molecules. Then the energy change was obtained by subtracting the sum of the static energy of nine molecules of water and one β-cyclodextrin molecule with the optimized initial configuration shown in section 2. When the nine water molecules contacted with the cavity side of β-cyclodextrin, as shown in Fig. 6(b), some of the water molecules entered the cavity and others stayed on the cavity side of β-cyclodextrin^27^ based on molecular dynamics (step interval: 2.0 fs, frame interval: 10 fs, terminate after 10 000 steps, heating/cooling rate: 1.0 kcal per atom per ps, and target: 313 Kelvin) six times. For these six times, the optimized total energy based on the minimum energy model fitting after molecular dynamics varied in the range of −67.1 to −71.9 kJ mol^−1^. When the nine water molecules contacted with the cavity toroid section of β-cyclodextrin, as shown in Fig. 6(c), the optimized total energy based on minimal energy model fitting after molecular dynamics varied in the range of −72.7 to −75.9 kJ mol^−1^, together with the spatial configurations shown in Fig. 6(e). Compared with the enthalpy change of 62.9–71.6 kJ mol^−1^ (water) during dehydration in section 3.6, it can be concluded that most water molecules contacted and interacted with β-cyclodextrin from its cavity side. Then these water molecules may play act as a bridge among the β-cyclodextrin molecules.

**Fig. 7:**
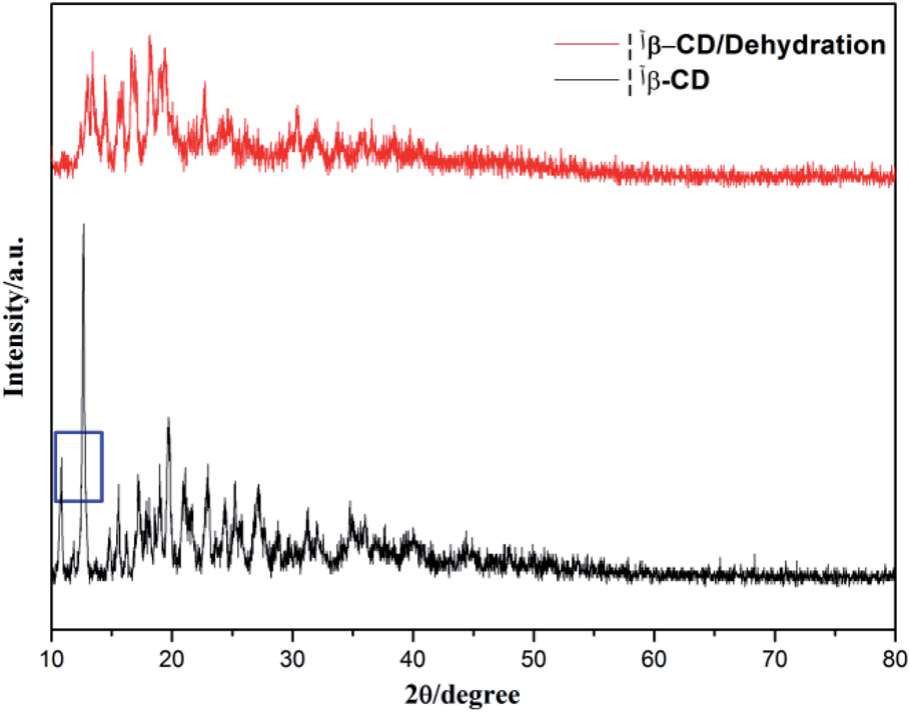
XRD patterns of β-cyclodextrin before and after water desorption.

To verify this, the X-ray diffractions of β-cyclodextrin before and after water desorption were obtained, as shown in Fig. 7, where in the XRD pattern, the peak at 2*θ* (10.8) disappeared after water desorption. Meanwhile, the intensity of the peak at 2*θ* (12.7) weakened and shifted after water desorption. This indicates that the water molecules should participate in the formation of the planar structure of β-cyclodextrin.

## 4. Conclusions

The hygroscopicity of β-cyclodextrin was investigated based on the dynamic adsorption method, TG-DSC analysis, and molecular dynamic simulation. The findings led to the comprehensive analysis of the adsorption characteristics of water on β-cyclodextrin. The results demonstrated that the system state of water adsorption on β-cyclodextrin depends on the amount of water adsorbed besides the adsorption temperature and vapor pressure of water, and the interaction between β-cyclodextrin and water is heterogeneous with an increase in the amount of water adsorbed or desorbed. Water adsorption on β-cyclodextrin is much easier than water desorption. Some interesting phenomena about the intermolecular interactions between β-cyclodextrin and water such as the adsorption rate, adsorption heat, activation energy and enthalpy change were found, which have rarely been reported before.

## Conflicts of interest

There are no conflicts to declare.

## Supplementary Material
